# An assay for chemical nociception in *Drosophila* larvae

**DOI:** 10.1098/rstb.2019.0282

**Published:** 2019-09-23

**Authors:** Roger Lopez-Bellido, Nathaniel J. Himmel, Howard B. Gutstein, Daniel N. Cox, Michael J. Galko

**Affiliations:** 1 Department of Genetics, University of Texas MD Anderson Cancer Center, Houston, TX 77030, USA; 2 Department of Anesthesiology, University of Pittsburgh, Pittsburgh, PA 15261, USA; 3 Neuroscience Institute, Georgia State University, P.O. Box 5030, Atlanta, GA 30303, USA; 4 Neuroscience Graduate Program, The MD Anderson UT Health Graduate School of Biomedical Sciences, TX 77030, USA; 5 Genetics and Epigenetics Graduate Program, The MD Anderson UT Health Graduate School of Biomedical Sciences, TX 77030, USA

**Keywords:** *Drosophila*, chemical nociception, sensory neurons, Basin interneurons, sensitization, allodynia

## Abstract

Chemically induced nociception has not yet been studied intensively in genetically tractable models. Hence, our goal was to establish a *Drosophila* assay that can be used to study the cellular and molecular/genetic bases of chemically induced nociception. *Drosophila* larvae exposed to increasing concentrations of hydrochloric acid (HCl) produced an increasingly intense aversive rolling response. HCl (0.5%) was subthreshold and provoked no response. All classes of peripheral multidendritic (md) sensory neurons (classes I–IV) are required for full responsiveness to acid, with class IV making the largest contribution. At the cellular level, classes IV, III and I showed increases in calcium following acid exposure. In the central nervous system, Basin-4 second-order neurons are the key regulators of chemically induced nociception, with a slight contribution from other types. Finally, chemical nociception can be sensitized by tissue damage. Subthreshold HCl provoked chemical allodynia in larvae 4 h after physical puncture wounding. Pinch wounding and UV irradiation, which do not compromise the cuticle, did not cause chemical allodynia. In sum, we developed a novel assay to study chemically induced nociception in *Drosophila* larvae. This assay, combined with the high genetic resolving power of *Drosophila,* should improve our basic understanding of fundamental mechanisms of chemical nociception.

This article is part of the Theo Murphy meeting issue ‘Evolution of mechanisms and behaviour important for pain’.

## 1. Introduction

All animals can discriminate noxious environmental stimuli that can potentially induce tissue damage. This nociceptive sensory capacity is crucial for health and survival. Noxious thermal, mechanical or chemical stimuli elicit specific escape behaviours designed to avoid the potentially damaging stimulus [1]. *Drosophila* larvae display different nociceptive responses based on the stimulus presented. Noxious heat and harsh mechanical stimuli induce bending [2], rolling and fast crawling behaviours [3,4], with rolling representing the dominant response. Conversely, noxious cold provokes a different behaviour, contraction of the head and tail towards the middle of the body [5]. Assays that measure aversive behaviours are helping to dissect the fundamental mechanisms of thermal [4–6] and mechanical nociception [7–9].

Chemical nociception has been relatively understudied in most organisms. In adult flies TRPA1, an evolutionarily conserved cation channel expressed in gustatory neurons, mediates chemical avoidance to noxious compounds such as aristolochic acid [10], allyl isothiocyanate (AITC, the noxious compound found in wasabi) and benzyl isothiocyanate (BITC) [11], *N*-methyl maleimide (NMM) and cinnamaldehyde (CA) [12]. These studies have measured proboscis extension to potential food sources rather than whole-animal aversion to chemicals. As such they examine an intersection between gustation (taste) and chemical nociception. Assays that probe responses that might be evoked by contact of noxious chemicals with the skin barrier have not yet been developed in *Drosophila*. What kind of aversive response might result from exposure to noxious chemical(s) is currently unknown.

*Drosophila* larvae have two major types of peripheral sensory neurons, type I and type II [13]. Type I neurons have a single ciliated dendrite that mediates mechanosensory functions such as light touch [14]. Type II neurons, also called multidendritic (md) neurons, exhibit elaborate dendritic projections that cover the barrier epidermis [15]. The md neurons comprise four classes (classes I–IV) based on their peripheral arbour complexity [16]. Class I neurons have been implicated in locomotion and proprioception [17–20]. Both class II and class III have been implicated in light-touch responses, with class III playing the major role [21,22]. Cold nociception is also mediated by class III neurons [5]. Class IV neurons are multimodal nociceptors, implicated in behavioural responses to noxious heat [4], harsh mechanical stimuli [9] and noxious low wavelength light [23]. At present, it is unknown which, if any, peripheral sensory neurons have a role in chemical nociception.

Second-order interneurons located in the ventral nerve cord receive synaptic input from the various classes of md sensory neurons [24]. These second-order interneurons likely allow integration of different sensory stimuli [2,7,25–27]. Basin interneurons (Basins 1–4) were the first identified interneurons implicated in multisensory integration and noxious responses [25,26]. Basin-1 mediates mechanosensory responses (vibration), while Basin-4 regulates thermal nociceptive responses via Goro neurons [26]. Other classes of second-order neurons, including medial clusters of class IV dendrite arborization (C4 da) second-order interneurons (mCSIs) [27], Down-and-Back (DnB) interneurons [2], dorsal pair (DP) insulin-like peptide 7 (ilp7) producing neurons (DP-ilp7 neurons) [7] and A08n neurons [7,28], also regulate nocifensive behavioural responses in *Drosophila* larvae. The roles, if any, of these different interneurons in chemical nociception have not yet been elucidated.

Tissue injury typically causes nociceptive sensitization [29]. For instance, UV exposure in *Drosophila* larvae sensitizes nociceptive sensory neurons to an innocuous thermal stimulus (thermal allodynia) [30]. This assay has helped to identify several signalling pathways mediating thermal allodynia, including TNF [30,31], Hedgehog [32] and tachykinin signalling [33]. In vertebrates, tissue damage can also cause hypersensitivity to chemical stimuli. A classic example would be the sting resulting from lemon juice when it encounters an open cut on a cook's hand. Whether chemical nociceptive responses in *Drosophila* can be sensitized by tissue injury remains an open question.

## 2. Results

### (a) Development of a novel assay for chemical nociception in *Drosophila* larvae

**Figure 1.**
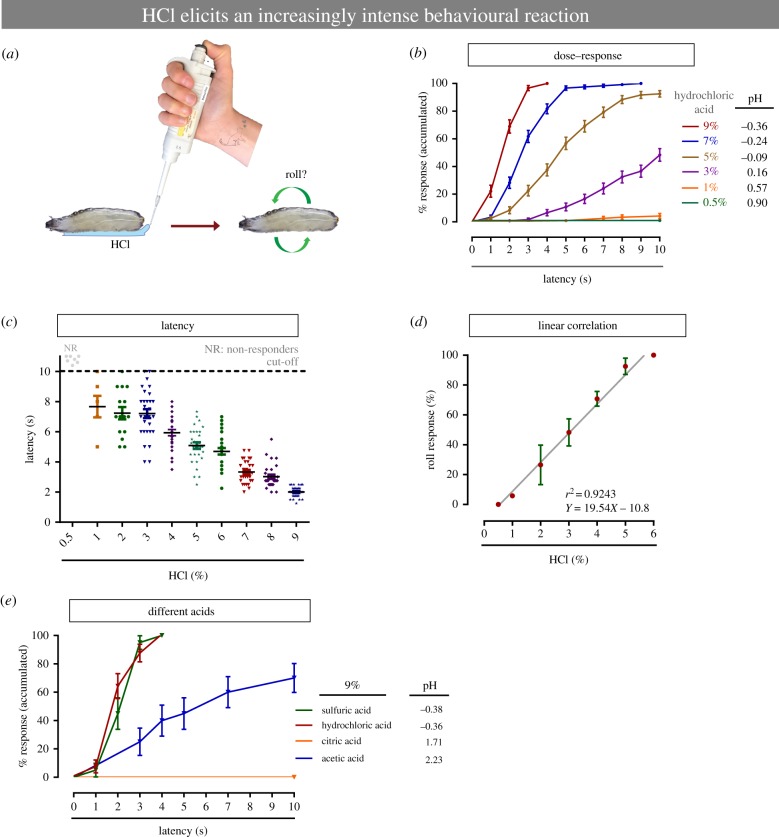
Development of a novel chemical nociception assay in *Drosophila* larvae. (*a*) Schematic cartoon of chemical nociception assay. (*b*) Behavioural aversive rolling response versus increasing chemical stimulus concentrations. (*c*) Latency of the behavioural response. (*d*) Relationship between chemical concentration and nociceptive behaviour. (*e*) Behavioural aversive rolling response to different acids. (*b–d*) *n* = 30 larvae and repeated three times. (*e*) 20–30 larvae. Error bars represent mean ± s.e.

To study chemical nociception in *Drosophila* larvae we developed a new behavioural assay. In this procedure, *Drosophila* larvae are briefly exposed to HCl, a presumably noxious acid (figure 1*a*). Concentrations below 0.5% HCl did not elicit responses that differed from normal locomotion (electronic supplementary material, movies S1 and S2); thus this concentration can be considered as subthreshold. Increasing the concentration of HCl (from 1 to 9%) caused aversive rolling, a behaviour that is also seen with exposure to noxious heat and mechanical stimuli [3,4,9] (electronic supplementary material, movies S3 and S4). This nociceptive behaviour was more prevalent as the acid concentration was increased (figure 1*b*) and the latency (time to rolling after HCl application) decreased dramatically with increasing acid concentration (figure 1*c*). The relationship between HCl concentration and behavioural responsiveness is linear (figure 1*d*). We also asked if other acids induce nociceptive behaviour at similar concentrations to HCl. Sulfuric acid induced a per cent of rolling response (100%) similar to HCl, while acetic acid was less potent (figure 1*e*). Among acids with slightly higher pH values, citric acid (pH 1.71) did not elicit any nociceptive behavioural responses, whereas acetic acid (pH 2.23) did (figure 1*e*). Larvae of different developmental stages did not show significant differences in nociceptive responsiveness to acid (*p* = 0.4667). However, larvae at the latest developmental stage (late third instar) did show a slight delay in the behavioural response when compared with the early developmental stages (electronic supplementary material, figure S1A). Together these data introduce and describe a new assay to measure chemical nociceptive behaviour in *Drosophila* larvae.

### (b) Noxious chemical stimulation induces cellular stress and tissue damage

**Figure 2.**
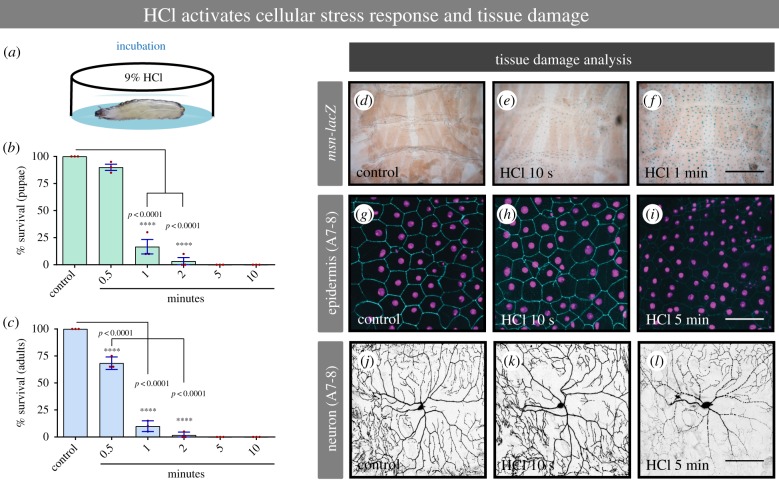
Noxious chemical stimulus induces cellular stress response and tissue damage. (*a*) Cartoon depicting the larvae incubation in 9% HCl. (*b,c*) Larval and adult survival per cent in response to increasing time of incubation with 9% HCl. (*d–f*) Epidermal cell stress response induced by 9% HCl. (*g–i*) Epidermal tissue damage analysis in response to noxious acid. (*j–l*) Class IV sensory neuronal tissue damage in response to noxious acid. A7-8: Abdominal segments 7 or 8. *msn-lacZ*: reporter of Jun N-terminal kinase (JNK) signalling/cellular stress. (*b,c*) *n* = 60 for each condition. Error bars represent mean ± s.e. Statistical significance was determined via one-way ANOVA with a Dunnet's *post hoc* test. (*d–f*) *n* = 4 larvae for each condition; (*g–l*) *n* = 9 larvae for each condition and representative images are shown. Bar in *d–f*, 50 µm. Bar in *g–l*, 100 µm.

Noxious stimuli evoke an aversive behavioural reaction presumably because they cause tissue damage and/or adversely affect survival. We tested whether exposure to acid is noxious by these criteria. Exposure to a high concentration of HCl (figure 2*a*, 9%) for increasing times dramatically decreased the survival rate of larvae to the pupal and adult stages (figure 2*b,c*). We next examined whether HCl exposure caused a cell stress response in third instar larvae. We exposed larvae to 9% HCl for either 10 s or 1 min and examined activation of *msn-lacZ*, a reporter of Jun N-terminal kinase (JNK) signalling/cellular stress [34]. We observed a modest induction of JNK activity in the barrier epidermal sheet upon brief exposure to 9% HCl when compared with the control larvae (figure 2*d–f*). At the cellular level, reduction of an epidermal membrane marker (figure 2*g–i*) and blebbing along the neuronal dendritic branches of class IV nociceptive sensory neurons were also observed (figure 2*j–l*). These changes were a function of both time of acid exposure and proximity to the posterior location of initial acid exposure. The more posterior abdominal segments (A7-A8) were affected more severely than more anterior segments further from the initial exposure to acid (A4-A5; electronic supplementary material, figure S2A–F). Together, our results suggest that HCl exposure is noxious, as defined by a decrease in survival and qualitative indicators of tissue damage, and that the level of noxiousness is determined by time of exposure and proximity to the source of exposure.

### (c) Multiple classes of multidentritic sensory neurons mediate chemical nociception responses

**Figure 3.**
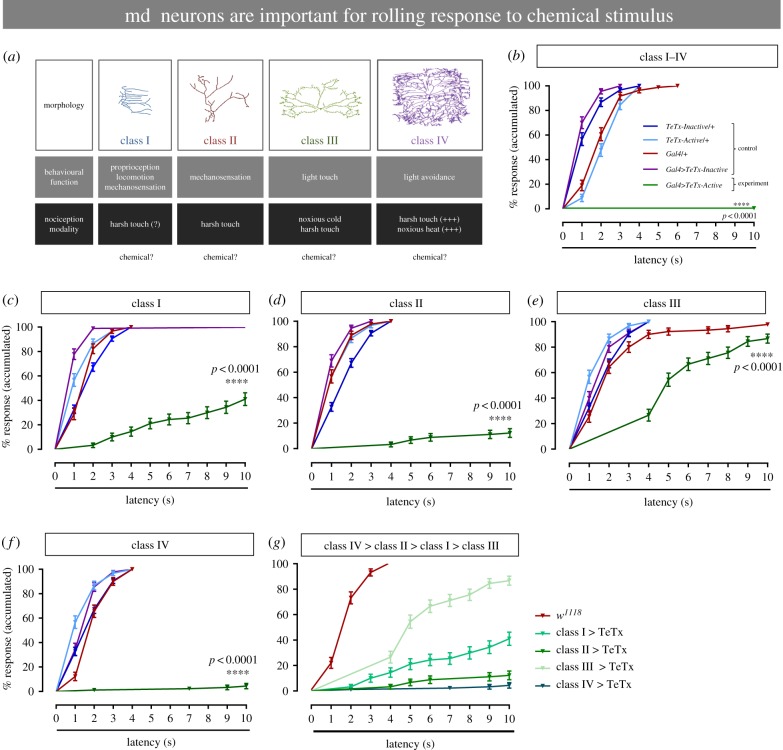
Multidentritic sensory neurons mediate chemical behavioural nociception responses. (*a*) Cartoon depicting the morphology, behavioural function, and nociceptive modality of the different peripheral sensory neurons. (*b–g*) Behavioural response to noxious chemical stimulus (HCl 9%). (*b*) Silencing of all multidendritic sensory neurons, by blocking the synaptic output using tetanus neurotoxin. Silencing specific sensory neurons using tetanus neurotoxin in class I (*c*), class II (*d*), class III (*e*) and class IV (*f*). (*g*) Comparison of the degree of the silencing effect in the md sensory neurons (classes I–IV). The genotype colour-code used in (*b*) applies for (*b–f*). (*b–f*) *n* = 90 for each condition, data from three independent replicates of at least 30 larvae each. Error bars represent mean ± s.e. Log-rank (Mantel–Cox) test was used to determine statistical significance between different groups.

The different classes of md sensory neurons (I–IV) that tile the larval barrier epidermis sense several sensory modalities, including heat, light touch, cold and harsh touch (figure 3*a*). Using a genetic inactivation strategy, we next asked whether md sensory neurons mediate chemical nociception. We combined a pan-md sensory neuron-specific Gal4 driver (*md-Gal4*) with a transgene encoding an active tetanus toxin (*UAS-TeTxLC* *=* *UAS-TeTx-Active*) or an inactive toxin control (*UAS-IMP TNT VI-A* *=* *UAS-TeTx-Inactive*) [35]. Expression of tetanus toxin in all md sensory neurons completely abrogated the response to a noxious chemical stimulus compared with relevant genetic controls (figure 3*b*). This suggests that one or more type of md sensory neuron class mediates chemical nociception.

We next silenced each class of md sensory neuron (I–IV) individually by combining sensory neuron class-specific Gal4 drivers with the active and inactive tetanus toxins used above. Since aversive rolling is seen with heat, harsh touch and chemical exposure we suspected that class IV sensory neurons might also play a role in chemical nociception. Surprisingly, silencing class I, class II, and to a lesser degree class III sensory neurons substantially attenuated chemical nociception (figure 3*c–e*). However, the strongest block of chemical nociception, as expected from the rolling behaviour, was seen with silencing class IV neurons (figure 3*f*). The strength of the nociceptive defect observed upon silencing each sensory neuron was class IV > class II > class I > class III (figure 3*g*). A separate class of peripheral sensory neurons, chordotonal neurons [16], had a minor effect on chemical nociceptive responses (electronic supplementary material, figure S3). Taken together, these data indicate that there is a distributed requirement for acid-evoked rolling in *Drosophila* larvae, with multiple md sensory neuron classes playing a role, either directly or indirectly, in the behavioural response.

### (d) Acid activates class IV nociceptive neurons

**Figure 4.**
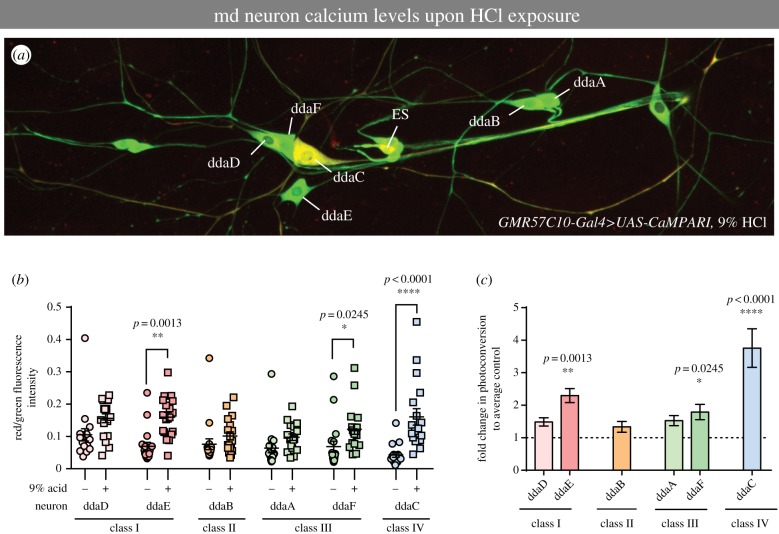
Acid activates class IV nociceptive neurons. (*a*) Representative image of CaMPARI photoconversion in the larval dorsal cluster of md sensory neurons following treatment with 9% HCl and violet light exposure. The class IV ddaC neuron visibly exhibits the highest level of red fluorescence relative to other md neurons subclasses/subtypes (ddaA/B/D/E/F). External sensory neuron (ES) also displayed relatively high fluorescence levels. (*b*) Red/green CaMPARI fluorescence intensity quantification for each md neuron class/subtype in control mock-treated (no HCl) or 9% HCl-treated third instar larvae. Each circle/square represents an individual neuron of the specified class. (*c*) Fold difference in green-to-red CaMPARI photoconversion by md neuron class/subtype when compared with mean vehicle control. (*b,c*) Data are means ± s.e.; *n* = 18 neurons; asterisks (*) indicates significant difference in CaMPARI photoconversion when compared with mock-treated vehicle control. Statistical significance between vehicle control and acid groups was determined via Kruskal–Wallis with Dunn's multiple comparisons test.

As multiple classes of md sensory neurons exhibit differing contributions to HCl-induced chemical nociception, we next sought to assess how HCl exposure influenced neural activity in these neurons. Freely behaving third instar larvae expressing the genetically encoded calcium indicator CaMPARI [36] were subjected to treatment with 9% HCl and immediately analysed *post hoc* for neural activation in md neuron subclasses. As inactivation of md neurons was sufficient to inhibit acid-evoked rolling behaviour, we focused our analyses on CaMPARI responses in md neuron subclasses. Visually, this analysis revealed strong HCl-induced activation of class IV (dorsal dendritic arborization neuron C, ddaC) nociceptive neurons relative to mock-treated controls (figure 4*a*). When we quantified red/green fluorescence ratios in representative images, we observed HCl-induced activation of a class IV neuron (ddaC) and to a lesser extent a subset of class I (ddaE) and class III (ddaF) md neurons (figure 4*b,c*). No significant activation, relative to controls, was observed for the class II (ddaB) neurons (figure 4*b,c*), although there was an upward trend in CaMPARI photoconverted signal for all md neurons. These data support a primary sensory role for class IV neurons in chemical nociception.

### (e) Basin interneurons mediate chemical nociception

**Figure 5.**
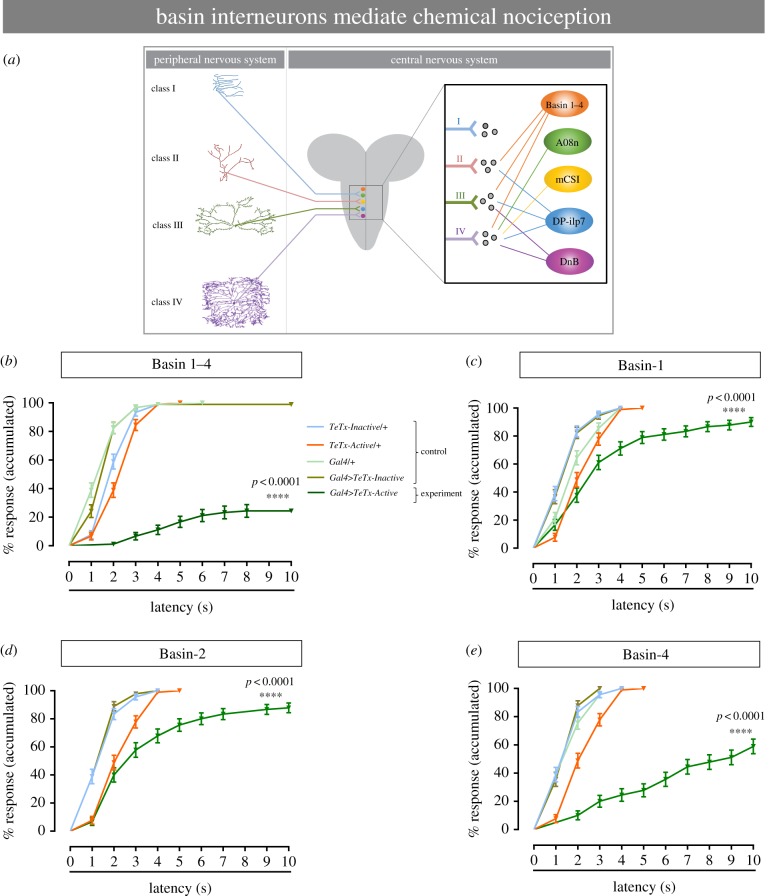
Second-order interneurons mediate chemical nociception response. (*a*) Schematic of the different peripheral sensory neurons and interneurons located in the ventral nerve cord. Adapted from Chin & Tracey [24]. (*b–e*) Behavioural response to noxious chemical stimulus (HCl 9%). (*b*) Silencing all Basin interneurons (Basin-1, Basin-2, Basin-3 and Basin-4) by blocking the synaptic output using tetanus neurotoxin. Silencing specific Basin second-order interneurons by using tetanus neurotoxin: (*c*) Basin-1, (*d*) Basin-2 and (*e*) Basin-4. The genotype colour-code used in (*b*) applies for (*b–e*). (*b–e*) *n* = 90 for each group, data from three independent replicates of 30 larvae each. Error bars represent mean ± s.e. Log-rank test was used to determine statistical significance between different groups.

Recent investigations have identified multiple interneurons that receive input from class IV sensory neurons and help mediate *Drosophila* larval nocifensive escape behaviours in response to noxious thermal and mechanical stimuli [2,7,26–28] (figure 5*a*). We next asked whether any of these interneurons (Basins, Goro, mCSI, DnB and A08n) also play a role in chemical nociception. We silenced each of these interneurons using neuron-specific Gal4 drivers (*Basin-Gal4*, *Goro-Gal4*, *mCSI-Gal4*, *DnB-Gal4* and *A08n-Gal4*) and the same tetanus toxin transgenes used above to silence peripheral sensory neurons. Ectopic expression of the active form of tetanus toxin transgene (*UAS-TeTx-Active*) in all Basin interneurons drastically reduced *Drosophila* larvae responses to 9% HCl, compared with the control larvae (figure 5*b*). This suggests that basin multisensory interneurons are required for the rolling behaviour response to chemical stimulus.

Basin interneurons comprise four neuron types of basin-shaped arbours (Basins 1–4), so we asked which of these interneuron types mediate chemical nociception using the same class-specific strategy we used for the sensory neurons, where class-specific drivers were available. When Basin-1 or Basin-2 interneurons were individually silenced, the nociceptive behavioural response to noxious chemical stimulation was partially decreased (figure 5*c,d*). A more significant decrease was observed when only Basin-4 neurons were silenced (figure 5*e*). The other interneuron classes (mCSIs, A08n and DnB) and neurons further in the nociceptive circuit (Goro) all displayed partial contributions to chemical nociception (electronic supplementary material, figures S4A–F), none of which was as strong as that observed by silencing Basins. Taken together, Basin interneurons, especially Basin-4 interneurons, appear to mediate chemical nociception.

### (f) Possible sensitization of chemical nociceptive responses by physical injury

**Figure 6.**
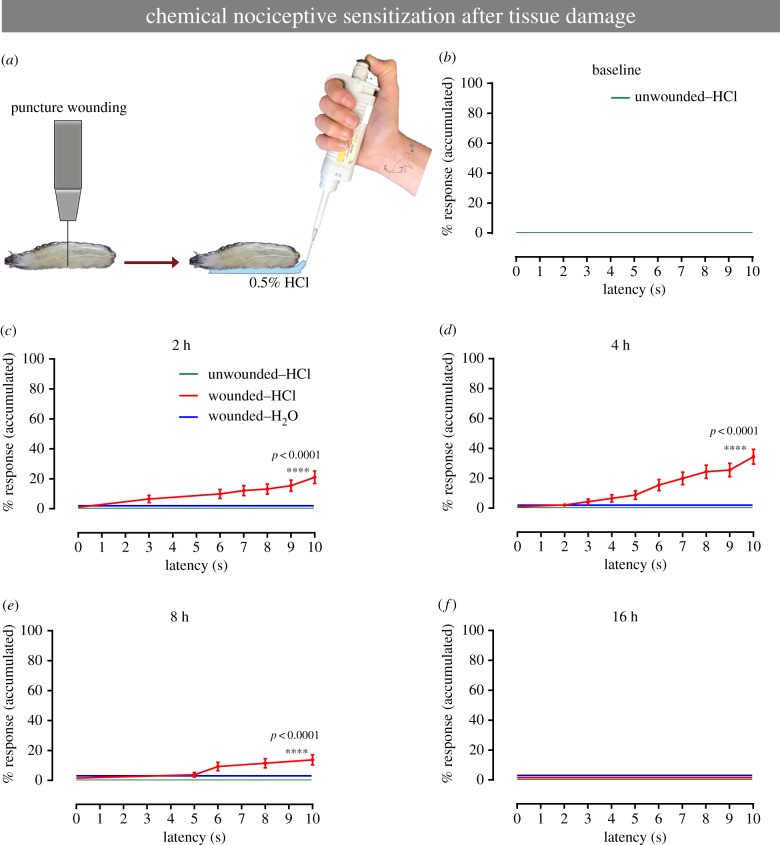
Chemical sensitization induced by tissue damage. (*a*) Schematic of chemical sensitization model. After the tissue damage, induced by puncture wound, animals were treated with non-noxious stimulus. (*b–f*) Behavioural response to non-noxious chemical stimulus (HCl 0.5%). (*b*) Baseline behavioural response of the animals to a benign solution, MilliQ water. (*c–g*) Behavioural response of the animals to non-noxious chemical stimulus, at different time points after puncture wounding: (*c*) 2 h, (*d*) 4 h, (*e*) 8 h and (*f*) 16 h. Colour-coding of groups used in (*c*) applies for (*c–f*). (*b–f*) *n* = 90 for each group, data from three independent replicates of 30 larvae each. Error bars represent mean ± s.e. Log-rank test was used to determine statistical significance between different groups.

A hallmark of nociceptive behaviours is that they can be sensitized by tissue injury, and this occurs with responses to both noxious heat [30] and noxious cold [37] in *Drosophila* larvae. Thus, we examined whether aversive rolling to a subthreshold concentration of HCl (0.5%), which would constitute chemical allodynia, could be observed in larvae. The classical sensitizing injury used in larvae to date is UV irradiation, which causes epidermal death and morphological disruption [30]. Very little responsiveness to subthreshold HCl was observed after this injury (electronic supplementary material, figure S5A,B). We reasoned that sensitization to chemical stimuli might require an injury that breaches the cuticle so we tried puncture wounding [34] (figure 6*a*). Compared with uninjured larvae (figure 6*b*), wounded larvae exhibited the emergence of an aversive rolling response at 2–4 h after injury (figure 6*c,d*). This acute hypersensitivity was short-lived, as it faded by 8 h post-injury (figure 6*e*) and was gone by 16 h post-injury (figure 6*f*). A breach in the cuticle appears to be important for the emergence of sensitization, as pinch wounding (which creates a larger wound but leaves the cuticle intact) [38] did not elicit chemical allodynia (electronic supplementary material, figure S5C–F). Taken together these results indicate that certain types of physical injuries that breach the cuticle may alter cuticle permeability in a manner that enhances the larval nociceptive response to a normally subthreshold concentration of HCl.

## 3. Discussion

Chemical nociception is arguably the least-studied noxious sensory modality to date in *Drosophila*. Ingestion (or potential ingestion) of noxious tastants such as AITC [12,39], aristolochic acid [10], cinnemaldehyde [12], l-canavanine [40,41] and acetic acid [42] reduces proboscis extension in *Drosophila* adults. Likewise, a volatile repellent, citronellal, induces an avoidance behaviour by activating olfactory receptor neurons [43]. These studies have identified a transient receptor potential (TRP) ion channel (TrpA1) and gustatory receptors that are required for inhibition of the proboscis extension response to potential food [10–12,39].

Our interest here was to develop an assay that challenges the barrier of the organism with a simple noxious chemical (in this case acid) and that does not necessarily have crosstalk with gustatory responses. Below, we discuss some of the implications of our findings with this new assay and their potential utility.

The md neuron(s) required for the full behavioural response to acid are distinct from larval gustatory neurons [44] and have not previously been implicated in taste. That md neurons are required for responses to acid is suggested by the fact that the observed behaviour (aversive rolling) is similar to that observed with noxious heat [4] and noxious mechanical stimuli [4,9]. Indeed, genetic silencing of class IV md neurons, which are also responsible for aversive rolling in response to temperatures above 40°C [4,30] and to harsh touch [4,8,9], led to a near-complete block of aversive rolling behaviour, suggesting that gustatory neurons have no role in the response to acid applied to the larval posterior. Surprisingly, similar class-specific silencing experiments revealed that all peripheral md neurons (class IV > class II > class I > class III) were required to some extent for acid-evoked rolling—not just the expected class IV neurons. Fast-rolling is only observed with optogenetic activation of class IV neurons [3,5], while optogenetic activation of classes I, II and III lead to other behaviours (halting or contraction/C-bend/slow-rolling) [5,7]. Our results add to the diverse array of modalities that can elicit rolling from class IV neurons (heat, harsh touch, noxious light) and raise questions about how the other classes are involved.

Our CaMPARI calcium imaging experiments indicated that class I and class III neurons were also activated by acid exposure, whereas class II was not. Because our genetic silencing experiments indicate that class II and III md neurons are also required for acid-evoked behaviour, these data suggest that class II and III neurons may facilitate rolling behaviour through downstream circuitry, rather than by direct activation by acid exposure. This seems possible as multiple classes of downstream second-order neurons were at least partially required for acid-evoked rolling. Furthermore, recent studies have demonstrated that class II, III and IV neurons share common postsynaptic interneuron partners implicated in rolling behaviour. For example, class II and IV neurons connect to DP-ilp7 interneurons to facilitate rolling in response to mechano-nociceptive stimuli [7], while class II, III and IV neurons connect to Basins [26]. These second-order neurons have been implicated in responses to other noxious sensory modalities [2,7,26], and, as we determined in this work, Basin 1–4 interneurons (mainly Basin-4) also mediate chemical nociception, suggesting these interneurons are multisensory integrators [25,26]. It will be interesting to determine how the full circuit(s) differ when a larva is challenged with particular stimuli.

In *Drosophila* thermal nociceptive sensitization has thus far been studied most extensively. Following UV irradiation both thermal allodynia (emergent responses to subthreshold stimuli) and thermal hyperalgesia (intensified responses to noxious stimuli) are observed [30]. By contrast, UV irradiation causes a behavioural switch from contraction to head and tail raising in response to noxious cold [37]. Curiously, neither UV irradiation nor physical pinch wounding (an injury that causes extensive damage to underlying sensory neurons) evoked chemical allodynia. Puncture wounding, however, a procedure that creates a breach in the overlying cuticle barrier, caused a transient (2–4 h) and relatively mild hypersensitivity to subthreshold acid. Whether the observed behavioural hypersensitivity represents a change in cuticle permeability that increases exposure to exogenously applied acid or is the result of specific damage-induced signalling pathways that alter neuronal firing is not yet clear. Moving forward, it will be interesting to test the involvement of previously identified thermal acute sensitization pathways [30,32,33], insulin signalling, which regulates the duration of thermal nociceptive sensitization [45], and Pvr signalling, which sets the threshold of mechanical nociception [46].

For humans, chemical nociception may be of less importance than the thermal or mechanical modalities as exposure to relevant environmental stimuli—sharp objects or extreme temperatures—are substantially more common than exposure to noxious acid. That said, local release of protons nearly always accompanies tissue damage, and thus acid-sensing may be a general feature of diverse injury-induced sensory responses [47]. Chemical nociception may be more important for *Drosophila* larvae as they may encounter relevant concentrations of acids, alcohols and other noxious chemicals as they feed. Although environmental exposure to harsh acids may not be common for fly larvae, the apparent pH threshold for responsiveness may be in the range that larvae experience in rotting or overcrowded fruit. The assay developed here has a simple practical advantage over thermal nociception assays [4,30] developed to date. Both thermal assays (hot and cold) require specialized tools capable of delivering a precise set temperatures to the larva. This can lead to some user-to-user variability in dose–response curves [30,31,33,48] and requires user expertise. Mechanical nociceptive assays developed to date [8,9] require a similar user competence. Chemical nociception is substantially easier to assess as it simply involves pipetting a small volume of solution on the larva and observing the resulting behaviour. *Drosophila* are well-suited for gene discovery [49] and the development of this chemical nociception assay enables future efforts to unravel the molecular/genetic basis of this understudied sensory modality.

## 4. Experimental procedures

### (a) *Drosophila* stocks and genetics

All fly stocks used in this work were maintained on cornmeal medium at 18°C*.* Larval progeny used in behavioural experiments were raised at 25°C. *w^1118^* was used as a control strain. *msn-lacZ* (*l(3)06946*) was used to monitor JNK activation [34]. The GAL4/UAS system was used to drive tissue-specific gene expression of transgenes under UAS control [50]. The following Gal4 lines were used: *Gal4^2-21^* (class I) [51], *GMR37B02-Gal4* (class II) [52], *19-12-Gal4* (class III) [23], *ppk1*.*9*-*Gal4* (class IV) [53], *md-Gal4^109(2)80^* (class I–IV) [54], chordotonal neurons *ch-Gal4* (BL59949) [55], Basin-Gal4 interneuron drivers (Basin 1–4, *GMR72F11-Gal4*; Basin-1, *JRC-R20B01-Gal4*; Basin-2, *RC-SS00739-Gal4*; and Basin-4, *JRC-SS00740-Gal4*); Goro interneurons (*R72F11-Gal4*) [26], mCSIs interneurons (*R94B10-Gal4,* referred to here as *mCSI-Gal4-1,* and *R52F05-Gal4,* referred to here as *mCSI-Gal4-2*): [27], *A08n-Gal4* interneurons (*GMR82E12-Gal4*) [7], DnB interneurons (*412-Gal4*, referred to here as *DnB-Gal4-1*, and *4051-Gal4,* referred to here as *DnB-Gal4-2*) [2]. *GMR57C10-Gal4* (pan-neuronal) and *Goro neuron-Gal4* (*GMR69F06-Gal4*) were obtained from Bloomington (BL39171 and BL39497, respectively). *e22c-Gal4, UAS-DsRed2Nuc(21),FasIII-GFP* [56] and *ppk-Gal4, UAS-mCD8-GFP* were used to label larval epidermal membranes and the class IV sensory neurons, respectively. The following UAS transgenes were used: *UAS-TeTxLC* (*UAS-TeTx-Active*) and *UAS-IMP TNT VI-A* (*UAS-TeTx-Inactive*) [35]; *UAS-CaMPARI* [36]. See electronic supplementary material, table S1 for a list of genotypes of flies used in each figure/panel.

### (b) Chemical nociception assay

A concentrated HCl stock solution (Sigma: 37.5–38.5%) was first diluted to 10% (assuming the stock concentration was 37%). The 10% stock was further diluted with MilliQ water to generate HCl solutions ranging from 0.5 to 9%. All of the other acids: sulfuric acid (vol/vol %), acetic acid (vol/vol %), and citric acid (wt/vol %) were used at 9% concentration. A pH meter (Accumet Basic, Fisher Scientific) was used to measure the pH, at room temperature, of the different acid solutions. Individual mid-third instar *Drosophila* larvae, crawling freely on 48-well cell culture plate covers made of polystyrene, were exposed to a particular solution by pipetting 1.5 µl of an HCl dilution (or MilliQ water for control) to the posterior end of the larva. Larvae were allowed to explore the dish for about 10 s before challenging them with the chemical stimulus. Larvae that reached the dish wall were redirected to the centre, before applying the chemical stimulus. If the pipette tip contacted the larva, that particular larva's behavioural response was not included to safeguard against the response being a combination of touch and acid. A complete roll of 360° along the body axis within 10 s of HCl exposure was scored as aversive behaviour. Other responses (fast crawling, turning, and bending) were not categorized as aversive responses for the purpose of this assay. When animals were exposed to a non-noxious or benign solution stimulus, MilliQ water, they did not elicit any reactions distinct from normal locomotion or light touch. Each larva was assessed behaviourally only once. The experimenter was blind to the genotype of the larva being tested. Three independent trials of 30 larvae each were performed unless otherwise indicated.

Behavioural data generated were plotted as the accumulated per cent (%) of response on the *Y*-axis versus latency (0 to 10 s) on the *X*-axis. A 100% response means all larvae exhibited an aversive roll (at some latency under the 10 s cut-off) and a 0% response means none exhibited an aversive roll at any latency under the 10 s cut-off. This method of plotting the frequency distribution of responses generates curves that are similar to lifespan curves for survival data and allows comparison of how many total larvae responded, how many did not respond, and how many responded at each particular latency. The log-rank Mantel–Cox test was used to statistically compare any curves plotted in this manner.

### (c) Immunofluorescence

To evaluate epidermal and neuronal tissue damage induced by HCl, third instar epidermal (*e22c-Gal4, UAS-DsRed2Nuc,FasIII-GFP*) and neuronal (*ppk-Gal4, UAS-mCD8-GFP*) reporter larvae were submerged in 100 µl of 9% HCl, anaesthetized with ether (ethyl ether anhydrous, Fisher Scientific), dissected in ice-cold phosphate-buffered saline (PBS), and then fixed for 1 h in 4% formaldehyde (FA). After several washes in PBS-Tx (1× phosphate-buffered saline with 0.3% Triton X-100) the samples were incubated overnight at 4°C with primary antibodies: mouse anti-GFP (1 : 500) for neurons; mouse anti-FasIII (1 : 50) and rabbit anti-DsRed (1 : 1000) for epidermal cells. Secondary antibodies (applied for 24 h at 4°C) were Alexa 647 anti-mouse (1 : 500) for neuronal samples and Alexa 488 anti-rabbit (1 : 500) and Alexa 647 anti-mouse (1 : 500) for epidermal tissues. After final washes in PBS-Tx, all stained samples were mounted in Vectashield (Vector Laboratories) and observed (see below).

### (d) β-Galactosidase histochemistry

Larvae carrying *msn-lacZ* were submerged completely in 100 µl of 9% HCl or MilliQ water for either 10 s or 1 min, incubated for 4 h at 25°C, dissected in ice-cold PBS, fixed for 30 min at room temperature with cold 2% glutaraldehyde, rinsed with PBS, and then stained at 30°C for 30–45 min with X-Gal (5-bromo-4-chloro-3-indolyl-d-galactopyranoside) as described [34].

### (e) Confocal microscopy and stereomicroscopy

Larvae were imaged on an Olympus FV1000 confocal microscope and Fluoview software was used to obtain the images. Laser wavelengths were 488 nm (Green Fluorescent Protein, GFP), and 543 nm (Far Red Fluorescent Protein). Images were captured at a resolution of 1024 × 1024 pixels for tissue damage experiments using a 20× numerical aperture (NA) 0.7 dry objective lens at 1.4× zoom. A *Z*-series stack, step-size of 1.5 µm, was collected and processed into a single *Z*-projection. Identical settings for laser intensity and other image capture parameters were applied for comparison of staining in the control and experimental groups. All figures were assembled with Photoshop CS6 and Illustrator CS6 (Adobe).

### (f) CaMPARI calcium analyses

For CaMPARI experiments, freely behaving third instar larvae expressing *UAS-CaMPARI* under the control of *GMR57C10-Gal4* were subjected to photoconverting light in the presence or absence (mock) of 9% HCl. Once larval locomotion initiated, 1.5 µl of either vehicle or 9% HCl was applied via micropipette to the posterior portion of the animal, and the subject was simultaneously illuminated with violet–blue, photoconverting light (excitation = 440 nm/short pass, dichroic mirror = 562 nm; Semrock) via a Zeiss AxioZoom V16 microscope, as previously described [5,57]. Larvae were left to behave under photoconverting light (84 000 lux) for 20 s. Dorsal clusters were then immediately imaged, *in vivo*, under a Zeiss LSM780 confocal microscope. *Z*-stacks were converted to maximum intensity projections, and *F*_red_ : *F*_green_ CaMPARI fluorescence intensity ratio was calculated using ImageJ software.

### (g) Induction of tissue damage

UV: Third-instar larvae were anaesthetized with ether then mounted ventral-side up on glass slides and placed in a Spectrolinker XL-1000 ultraviolet crosslinker (Spectronics Corporation). UV treatment lasted approximately 6 s and was approximately 14 mJ cm^−2^, at a wavelength of 254 nm. Larvae were returned to regular fly food at 25°C before measuring behaviour at various times after UV irradiation. Pinch and puncture wounding: These were performed as described previously [58].

### (h) Statistical analysis

All statistical analyses were performed using GraphPad Prism v. 7 (GraphPad Software, La Jolla, CA, USA, www.graphpad.com). All the data were tested for a normal distribution using Kolmogorov–Smirnov (KS) or Shapiro–Wilk normality test, and were then analysed using one-way ANOVA followed by Dunnet's *post hoc* test for survival assay. As calcium data were not normally distributed, differences were assessed by Kruskal–Wallis with Dunn's multiple comparisons test. For behavioural data, statistical significance was tested using log-rank (Mantel–Cox) analysis. Asterisks in the graphs indicate the significance of *p*-values comparing indicated group with controls: *p < 0.05, **p < 0.01, ****p < 0.0001.

## Supplementary Material

Figure S1. Chemical nociception at different stages of larval development.

## Supplementary Material

Figure S2. Tissue Damage Induced by Noxious Stimulus

## Supplementary Material

Figure S3. Chordotonal sensory neurons partially mediate chemical nociception

## Supplementary Material

Figure S4. Role of Second Order Interneurons in Chemical Nociception

## Supplementary Material

Figure S5. Chemical Sensitization induced by Tissue Damage

## Supplementary Material

Movie 1 (Normal Locomotion).mp4

## Supplementary Material

Movie 2 (Exposure to Water).mp4

## Supplementary Material

Movie 3 (Exposure to 5% HCl).mp4

## Supplementary Material

Movie 4 (Exposure to 9% HCl).mp4

## Supplementary Material

Supplementary Table 1 RE.docx
