# Supplementary figures and images for: An assay for chemical nociception in Drosophila larvae
Source: Philos Trans R Soc Lond B Biol Sci. 2019 Sep 23;374(1785):20190282. doi: 10.1098/rstb.2019.0282 (PMC6790381; doi:10.1098/rstb.2019.0282)

**Figure S1**

**Chemical nociception at different stages of larval development**

**A**

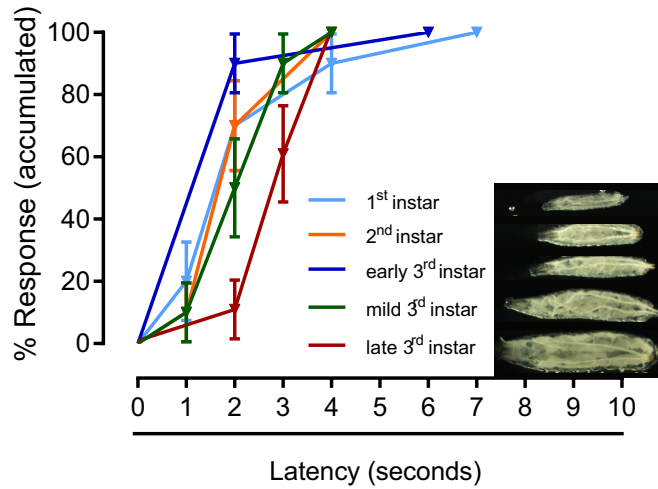

Supplement: Figure S1. Chemical nociception at different stages of larval development. [file rstb20190282supp1.pdf]

# Figure S2

## HCl induce cell stress and tissue damage

### Tissue damage analysis

Epidermis (A4-5)

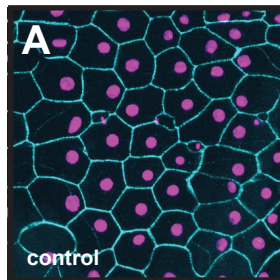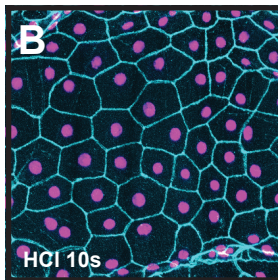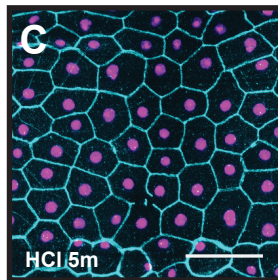

Neuron (A4-5)

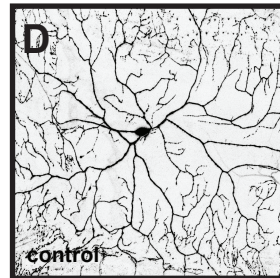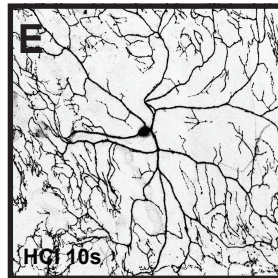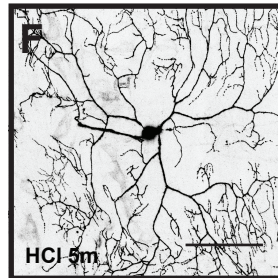

Supplement: Figure S2. Tissue Damage Induced by Noxious Stimulus [file rstb20190282supp2.pdf]

Figure S3

Chordotonal cells partially mediate chemical nociception

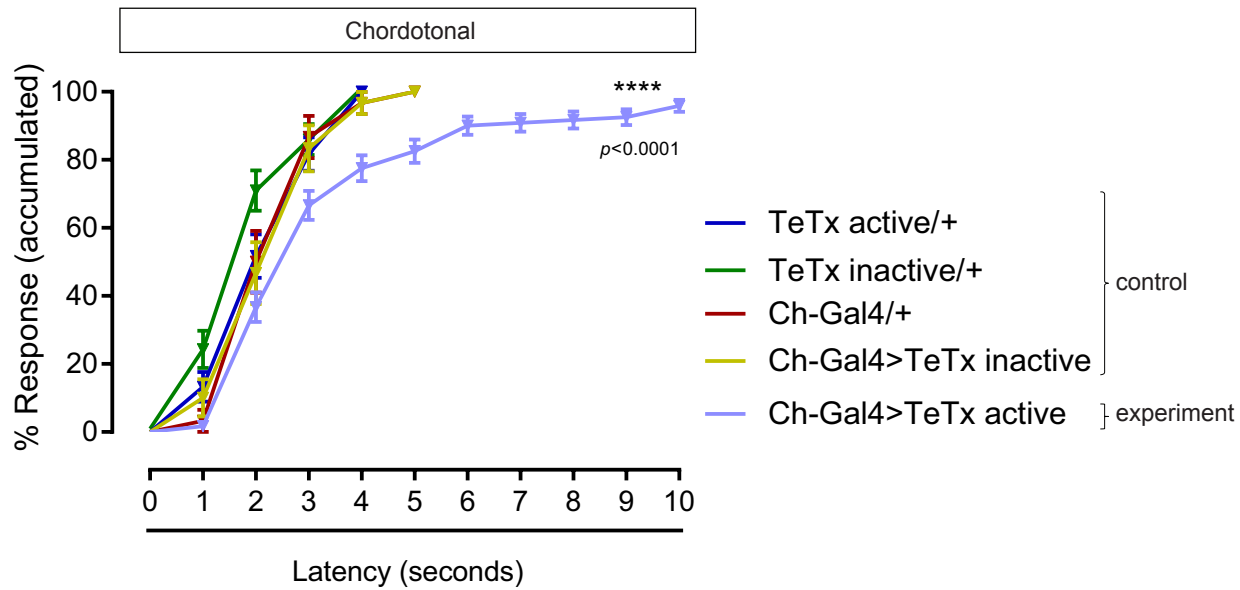

Supplement: Figure S3. Chordotonal sensory neurons partially mediate chemical nociception [file rstb20190282supp3.pdf]

**Figure S4****Contribution of other interneurons in chemical nociception**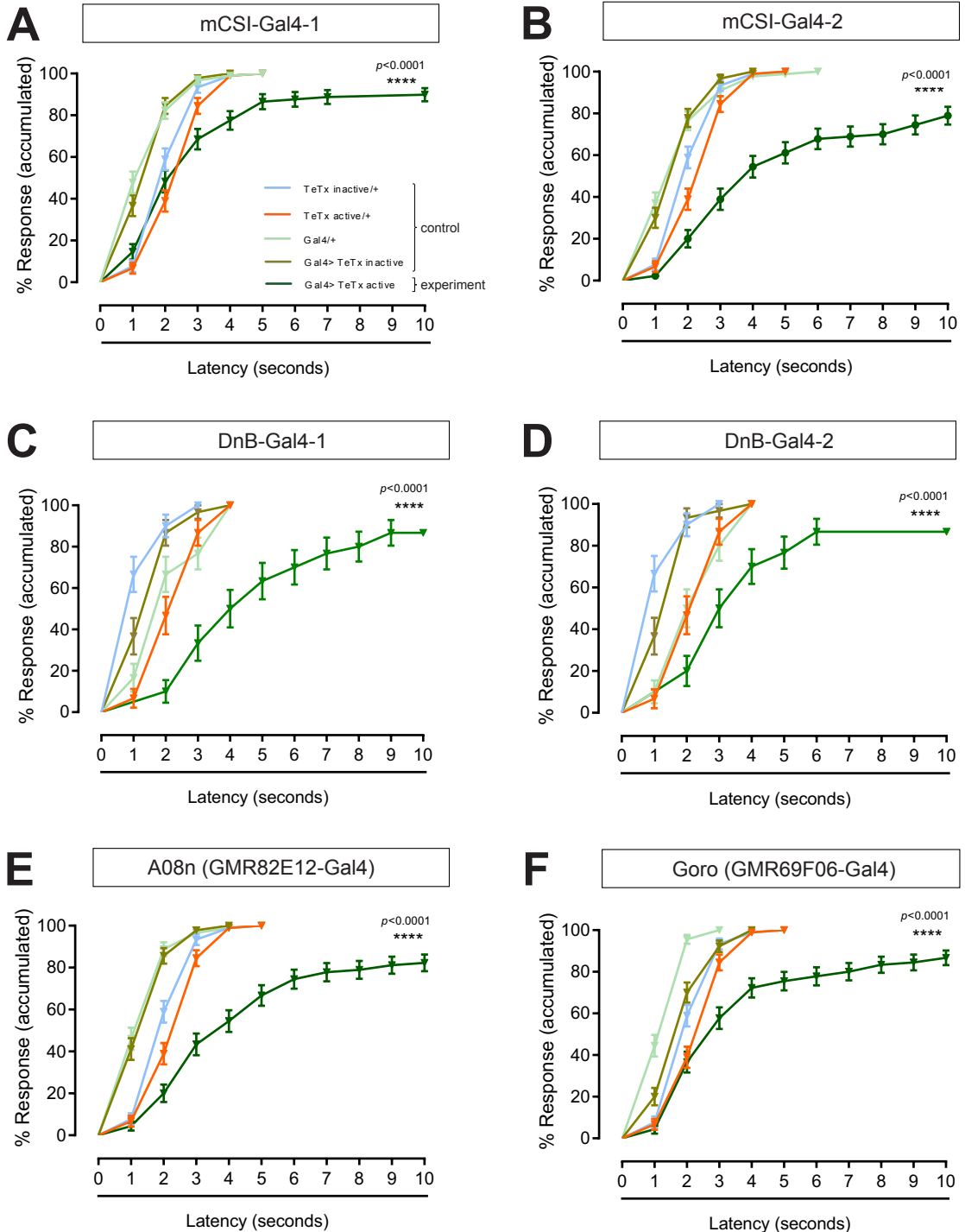

Supplement: Figure S4. Role of Second Order Interneurons in Chemical Nociception [file rstb20190282supp4.pdf]
