# Supplementary material for: An assay for chemical nociception in Drosophila larvae
Source: Philos Trans R Soc Lond B Biol Sci. 2019 Sep 23;374(1785):20190282. doi: 10.1098/rstb.2019.0282 (PMC6790381; doi:10.1098/rstb.2019.0282)
Supplement: Figure S5. Chemical Sensitization induced by Tissue Damage [file rstb20190282supp5.pdf]

Figure S5

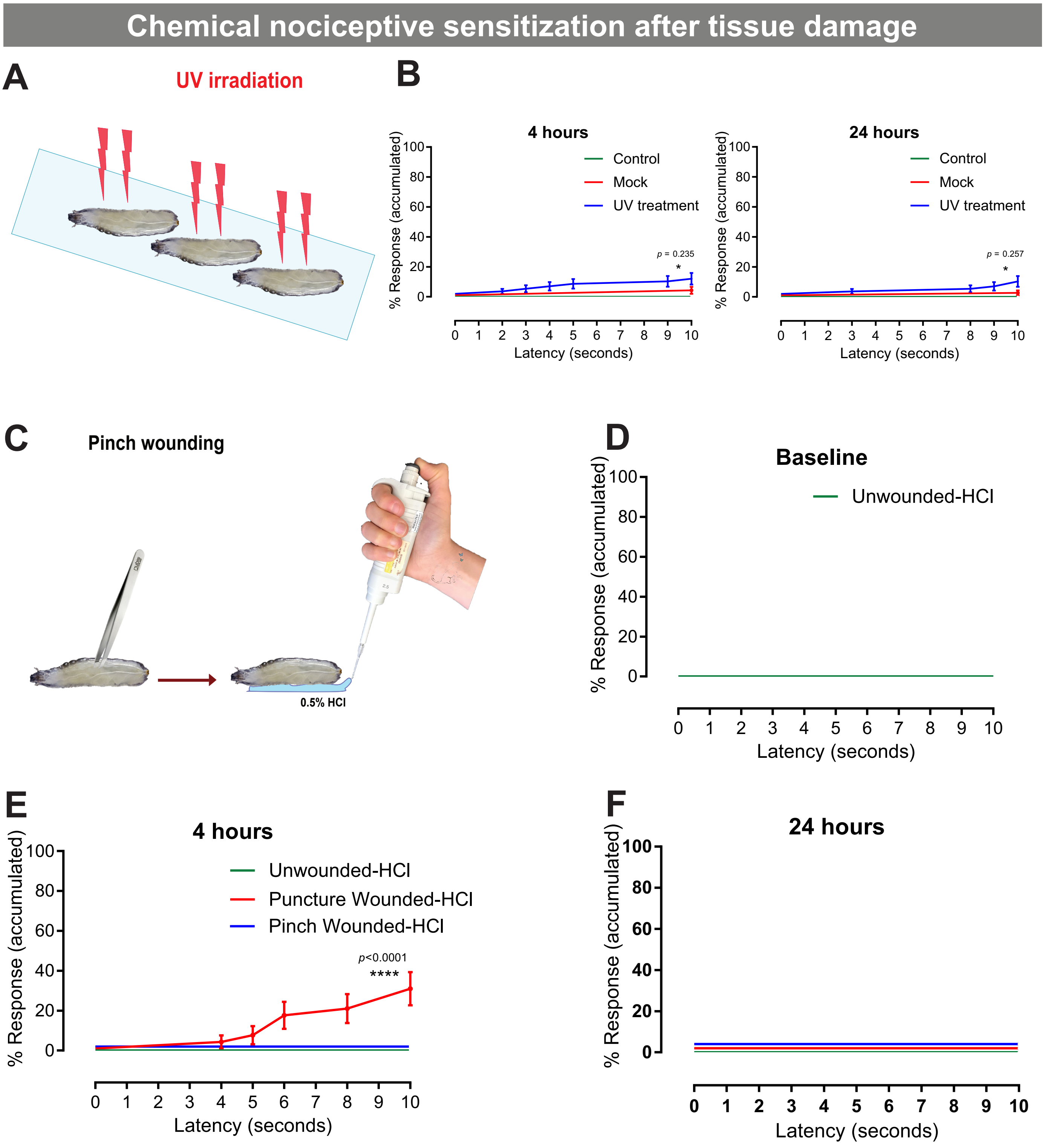
Chemical nociceptive sensitization after tissue damage
A
UV irradiation
B
4 hours
Control
Mock
UV treatment
p = 0.235
*
% Response (accumulated)
Latency (seconds)
24 hours
Control
Mock
UV treatment
p = 0.257
*
% Response (accumulated)
Latency (seconds)
C
Pinch wounding
0.5% HCl
D
Baseline
Unwounded-HCl
% Response (accumulated)
Latency (seconds)
E
4 hours
Unwounded-HCl
Puncture Wounded-HCl
Pinch Wounded-HCl
p<0.0001
****
% Response (accumulated)
Latency (seconds)
F
24 hours
% Response (accumulated)
Latency (seconds)
