# Supplementary material for: An assay for chemical nociception in Drosophila larvae
Source: Philos Trans R Soc Lond B Biol Sci. 2019 Sep 23;374(1785):20190282. doi: 10.1098/rstb.2019.0282 (PMC6790381; doi:10.1098/rstb.2019.0282)
Supplement: Supplementary Table 1 RE.docx [file rstb20190282supp10.docx]

**Supplementary Table 1. Genotypes used in this work.**

**Figure 1B-E** : *w^1118^*

**Figure 2B-C** : *w^1118^*

**Figure 2D-E** : *w^1118^;; msn-lacZ/+*

**Figure 2G-I** : *w^1118^; e22c-Gal4, UAS-DsRed2Nuc(2), FasIII-GFP*

**Figure 2J-L** : *w^1118^;; ppk-Gal4,UAS-mCD8::GFP/ppk-Gal4, UAS-mCD8::GFP*

**Figure 3B** : *w^1118^;UAS-TeTx Inactive/+*

*w^1118^;;UAS-TeTx Active/+*

*w^1118^;md-GAL4^109(2)80^/+*

*w^1118^; md-GAL4^109(2)80^/UAS-TeTx Inactive*

*w^1118^; md-GAL4^109(2)80^/+; UAS-TeTx Active/+*

**Figure 3C** : *w^1118^;UAS-TeTx Inactive/+*

*w^1118^;; UAS-TeTx Active/+*

*w^1118^;;Class I-Gal4/+*

*w^1118^; UAS-TeTx Inactive/+; Class I-Gal4/+*

*w^1118^;; Class I-Gal4/UAS-TeTx Active*

**Figure 3D** : *w^1118^; UAS-TeTx Inactive/+*

*w^1118^;; UAS-TeTx Active/+*

*w^1118^;; Class II-Gal4/+*

*w^1118^; UAS-TeTx Inactive/+; Class II-Gal4/+*

*w^1118^;; Class II-Gal4/UAS-TeTx Active*

**Figure 3E** : *w^1118^;UAS-TeTx Inactive/+*

*w^1118^;; UAS-TeTx Active/+*

*w^1118^;; Class III-Gal4/+*

*w^1118^; UAS-TeTx Inactive/+; Class III-Gal4/+*

*w^1118^;; Class III-Gal4/UAS-TeTx Active*

**Figure 3F** : *w^1118^; UAS-TeTx Inactive/+*

*w^1118^;; UAS-TeTx Active/+*

*w^1118^;; Class IV-Gal4/+*

*w^1118^; UAS-TeTx Inactive/+; Class IV-Gal4/+*

*w^1118^;; Class IV-Gal4/UAS-TeTx Active*

**Figure 4A-CF**: *w^1118^;UAS-CaMPARI/+;GMR57C10-GAL4/+*

**Figure 5B** : *w^1118^; UAS-TeTx Inactive/+*

*w^1118^;; UAS-TeTx Active/+*

*w^1118^;;Basin 1-4-Gal4/+*

*w^1118^; UAS-TeTx Inactive/+; Basin 1-4-Gal4/+*

*w^1118^;; Basin 1-4-Gal4/UAS-TeTx Active*

**Figure 5C** : *w^1118^; UAS-TeTx Inactive/+*

*w^1118^;; UAS-TeTx Active/+*

*w^1118^;; Basin-1-Gal4 /+*

*w^1118^; UAS-TeTx Inactive; Basin-1-Gal4 /+*

*w^1118^;; Basin-1-Gal4 /UAS-TeTx Active*

**Figure 5D** : *w^1118^; UAS-TeTx Inactive/+*

*w^1118^;; UAS-TeTx Active/+*

*w^1118^; Basin-2-Gal4 /+*

*w^1118^; Basin-2-Gal4 /UAS-TeTx Inactive*

*w^1118^; Basin-2-Gal4 /+;UAS-TeTx Active/+*

**Figure 5E** : *w^1118^; UAS-TeTx Inactive/+*

*w^1118^;; UAS-TeTx Active/+*

*w^1118^;* *Basin-4-Gal4 /+*

*w^1118^; Basin-4-Gal4 / UAS-TeTx Inactive*

*w^1118^; Basin-4-Gal4 /+; UAS-TeTx Active/+*

**Figure 6B-F** : *w^1118^*

**Figure S1A** : *w^1118^*

**Figure S2A-C**: *w^1118^; e22c-Gal4, UAS-DsRed2Nuc(21), FasIII-GFP*

**Figure S2D-F**: *w^1118^;; ppk-Gal4,UAS-mCD8::GFP/ppk-Gal4, UAS-mCD8::GFP*

**Figure S3** : *w^1118^;; TeTx Active/+*

*w^1118^; TeTx Inactive/+*

*w^1118^;; ch-Gal4/+*

*w^1118^; UAS-TeTx Inactive/+; ch-Gal4/+*

*w^1118^;; ch-Gal4/UAS-TeTx Active*

**Figure S4A** : *w^1118^; TeTx Inactive/+*

*w^1118^;; TeTx Active/+*

*w^1118^;; mCSI-Gal4-1/+*

*w^1118^; UAS-TeTx Inactive/+; mCSI-Gal4-1/+*

*w^1118^;; mCSI-Gal4-1/UAS-TeTx Active*

**Figure S4B** : *w^1118^; TeTx Inactive/+*

*w^1118^;; TeTx Active/+*

*w^1118^;; mCSI-Gal4-2/+*

*w^1118^; UAS-TeTx Inactive/+; mCSI-Gal4-2/+*

*w^1118^;; mCSI-Gal4-2/UAS-TeTx Active*

**Figure S4C** : *w^1118^; TeTx Inactive/+*

*w^1118^;; TeTx Active/+*

*w^1118^;;DnB-Gal4-1/+*

*w^1118^; UAS-TeTx Inactive/+; DnB-Gal4-1/+*

*w^1118^;; DnB-Gal4-1/UAS-TeTx Active*

**Figure S4D** : *w^1118^; TeTx Inactive/+*

*w^1118^;; TeTx Active/+*

*w^1118^;; DnB-Gal4-2/+*

*w^1118^; UAS-TeTx Inactive/+; DnB-Gal4-2/+*

*w^1118^;; DnB-Gal4-2/UAS-TeTx Active*

**Figure S4E** : *w^1118^; TeTx Inactive/+*

*w^1118^;; TeTx Active/+*

*w^1118^;; A08n-Gal4/+*

*w^1118^; UAS-TeTx Inactive/+; A08n-Gal4/+*

*w^1118^;; A08n-Gal4/UAS-TeTx Active*

**Figure S4F** : *w^1118^; TeTx Inactive/+*

*w^1118^;; TeTx Active/+*

*w^1118^;;Goro-Gal4/+*

*w^1118^; UAS-TeTx Inactive/+; Goro-Gal4/+*

*w^1118^;; Goro -Gal4/UAS-TeTx Active*

**Figure S5B-G**: *w^1118^*
